# Diagnostic delays and nuanced surgical management in perianal Paget’s disease: a case report and review of current literature

**DOI:** 10.1093/jscr/rjaf528

**Published:** 2025-07-14

**Authors:** Cyprien Jungels, Megan Bedard, Daine Bennett

**Affiliations:** HCA HealthOne, Swedish General Surgery Residency, Swedish Medical Center, 501 E Hampden Ave, Englewood, CO 80113, United States; HCA HealthOne, Swedish General Surgery Residency, Swedish Medical Center, 501 E Hampden Ave, Englewood, CO 80113, United States; SurgOne, PC, Colorectal Surgery, 8490 E Crescent Parkway, Suite 380, Greenwood Village, CO 80111, United States

**Keywords:** perianal Paget’s disease, wide local excision, chemotherapy, radiotherapy, multidisciplinary care

## Abstract

Perianal Paget’s disease is an uncommon and difficult to treat disease due to the high risk of positive margin at resection and high risk for recurrence. It can arise as a primary tumor or secondary to underlying pelvic visceral malignancy and is often initially misdiagnosed as an inflammatory perianal rash until proper pathologic diagnosis has been confirmed. We present the case of a 76-year-old woman who presented with 2 years of a perianal rash unsuccessfully treated with various topical interventions. Eventually, a biopsy was performed revealing extramammary Paget’s disease and she underwent surgical excision of the perianal lesion. Operative pathology unfortunately exhibited positive margins and we detail the multidisciplinary discussion of further management options in this complicated clinical dilemma. This case highlights the importance of early recognition of this disease and a multidisciplinary approach to its treatment.

## Introduction

Paget’s disease is a cutaneous malignancy most commonly seen in the breast. Perianal Paget’s disease is a rare disorder, and the available literature on the subject remains observational, with only a few retrospective studies published [1, 2].

Primary perianal Paget’s disease arises from epidermal cells with differentiation into apocrine glands. However, in some reviews of the literature, up to 42% of cases of perianal Paget’s disease were associated with underlying malignancies [3].

Unfortunately, the diagnosis is often delayed due to misdiagnosis and failed initial treatment as a benign dermatological condition.

## Case report

A 76-year-old woman presented with 2 years of a perianal rash previously evaluated by dermatology and wound care. Various topical interventions were attempted without improvement, and the lesion grew to a size of 5.0 × 3.7 cm over the course of 2 years. Physical examination revealed an erythematous macular lesion with intermittent ulceration over the left perianal skin and buttock. A biopsy was performed and histopathology revealed extramammary Paget’s disease involving the deep and lateral margins of the specimen, supported by a positive mucicarmine stain. Her medical history was significant for left breast cancer 20 years prior treated with mastectomy and hormonal therapy, with normal subsequent mammograms of the right breast.

**Figure 1:**
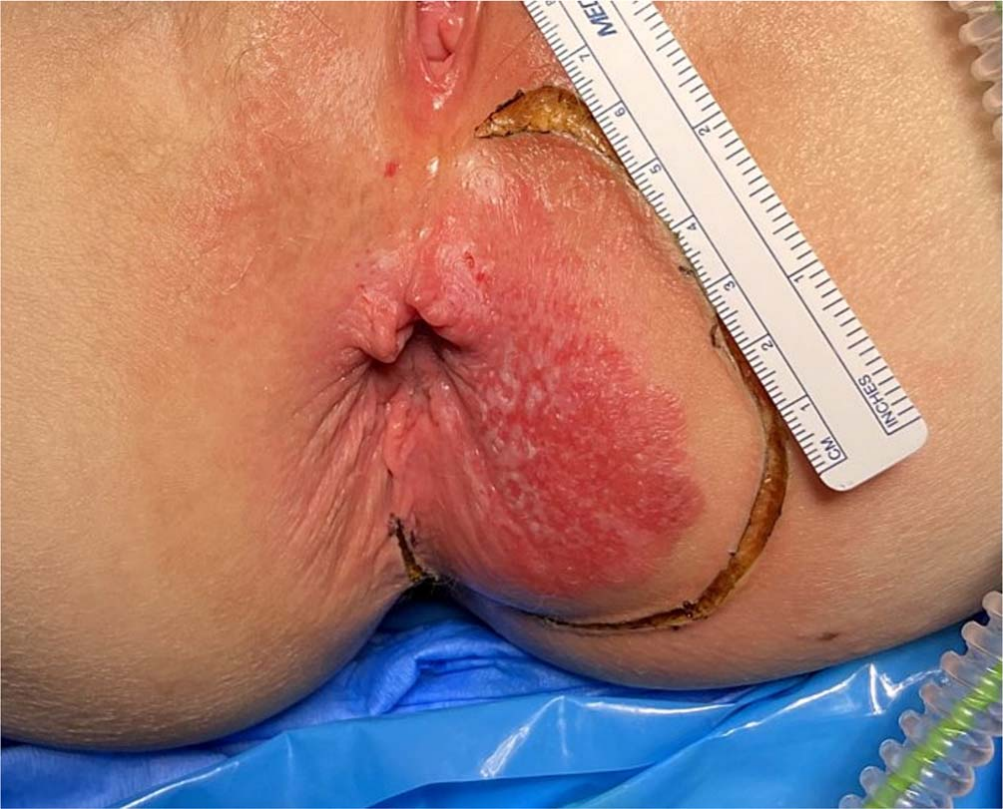
Perianal Paget’s disease prior to excision.

**Figure 2:**
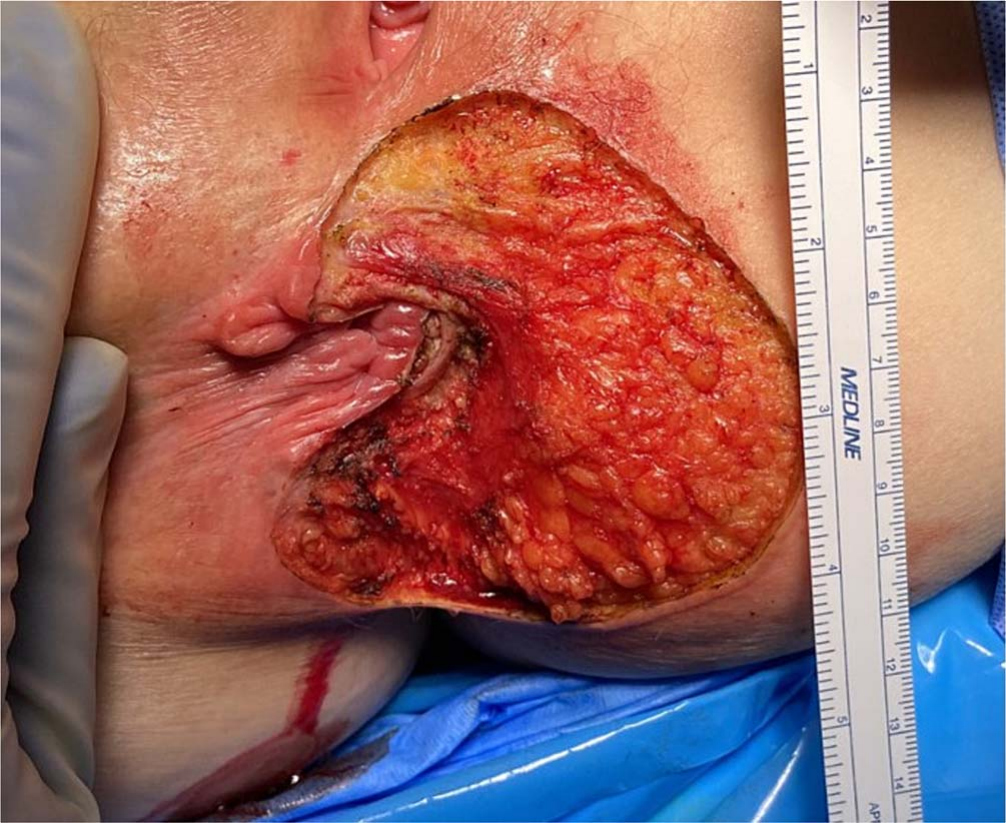
Perianal Paget’s disease after initial excision.

The preoperative workup included computed tomography of the chest, abdomen, and pelvis; positron emission tomography scan; magnetic resonance imaging of the pelvis, genetic testing, colonoscopy, cystoscopy, and Pap smear. No underlying malignancy was identified. She underwent surgical excision of the lesion with 1 cm margins and concomitant diverting colostomy given the high likelihood of fecal contamination of the surgical wound (Figs 1 and 2). Plastic surgery performed temporary wound coverage with allograft in the same procedure. Pathology was consistent with Paget’s disease and showed positive margins along the anal verge.

She was brought back for re-excision 3 weeks later given the positive margin. A further 1 cm margin was taken from the 6 to 12-o’clock (medial) position. Within the anal canal, <1 cm of epithelial tissue remained and the resection was taken to the dentate line for approximately one third of the anal canal. Plastic surgery performed repeat temporary wound coverage with allograft. Unfortunately, pathology again resulted with positive microscopic margins at the 12 and 6 o’clock positions.

Further treatment options were then discussed with the patient and her multidisciplinary team including dermatology, medical oncology, radiation oncology, dermatopathology, and colorectal surgery. Given her risk for fecal incontinence, the patient elected to forgo further excision. She underwent soft tissue flap coverage with plastic surgery. Adjuvant radiotherapy was offered but given personal concerns of effect on her quality of life, she declined further treatment and elected to proceed with expectant management with serial exams. Her ostomy was reversed 2 months after her flap reconstruction. She has had acceptable bowel function without incontinence since.

## Discussion

Paget’s disease is a cutaneous malignancy most commonly seen in the breast. It is a rare disorder, and the available literature on the subject remains observational, with only a few retrospective studies published [1, 2]. Primary Paget’s disease arises from epidermal cells with differentiation into apocrine glands. In more uncommon cases, perianal Paget’s disease can be found secondary to an underlying visceral malignancy, most commonly colorectal adenocarcinoma [4]. Up to 42% of cases were associated with underlying malignancies [3]. Unfortunately, the diagnosis is often delayed due to misdiagnosis and treatment as benign dermatological conditions.

Diagnosis is typically made on biopsy by histopathologic review, which can help differentiate between primary and secondary Paget’s disease. On histology, primary disease was associated with positive staining for cytokine 7 (CK7) and gross cystic disease fluid protein-15 (GCDFP-15), with negative staining of cytokine 20 (CK20) [5]. Perianal Paget’s disease secondary to underlying visceral neoplasm was found to be associated with positive staining for CK7 and CK20, and negative GCDFP-15 staining [5]. Due to the risk of associated underlying malignancy, further diagnostic workup is often required to rule out concomitant anorectal, adnexal, cervical, or urinary bladder adenocarcinoma [3, 6].

Treatment of perianal Paget’s disease, in the absence of colorectal involvement, depends on disease involvement of the anoderm. Typically, wide local excision with 1 cm margins is preferred [7]. The rate of positive margins is high at 34%–42% [2], and re-excision may need to be considered. A multidisciplinary approach with a plastic surgery team may be required for reconstruction after adequate excision is confirmed [8]. In the case of Paget’s disease encroaching on anoderm with inability to obtain adequate margins at the anal aspect of the specimen, an abdominoperineal resection (APR) may be considered. After excision, recurrence rates are 29%–61% [1, 2, 9–11]. Even after negative margins, recurrence rates of up to 33% have been described [12]. Frozen sections were considered for more accurate resection of all disease margins, but have largely been abandoned due to incorrect diagnosis rates of up to 37.5% [12].

Consideration can be made for adjuvant radiotherapy or chemotherapy on an individualized basis. In patients who are not surgical candidates, radiation therapy dosing at greater than 50 Gray (Gy) has been proposed, and adjuvant therapy at doses greater than 55 Gy is recommended in the case of concomitant adnexal or rectal adenocarcinoma [13]. Both topical and systemic chemotherapy for management of extramammary Paget’s disease have been described. Case series have described the use of topical 5-florouracil (5-FU), bleomycin, and imiquimod for both symptom control and cytoreduction [9]. Systemic regimens are typically recommended in patients who are not candidates for surgical excision or radiotherapy. These are often regimens containing systemic 5-FU used in combination with mitomycin C or carboplatin [9].

In conclusion, Perianal Paget’s disease can be a diagnostic and management challenge. Initial misdiagnosis is common and can delay appropriate treatment. Management itself can be complex as the rates of positive margins and recurrence after complete excision remain high. This underlines the importance of a multidisciplinary approach to care. Unfortunately, prognostic data remain scarce due to the limited literature on this disease, with 5-year overall survival rates of 59%–67% [11, 14].
